# Rapid weight gain in first 2 years of life and BMI trajectories from 3 to <10 years: a population-based longitudinal study of 1.7 million Brazilian children

**DOI:** 10.1016/j.lana.2025.101326

**Published:** 2025-12-03

**Authors:** Carolina Santiago-Vieira, Leah Li, Rita de Cássia Ribeiro Silva, Juliana Freitas de Mello e Silva, Enny S. Paixão, Maurício L. Barreto, Gustavo Velasquez-Melendez

**Affiliations:** a School of Nursing, Universidade Federal de Minas Gerais, Belo Horizonte, Brazil; b Center of Data and Knowledge Integration for Health (CIDACS), Fiocruz-Bahia, Salvador, Brazil; c Population, Policy and Practice Research and Teaching Department, Great Ormond Street Institute of Child Health, University College London, London, United Kingdom; d School of Nutrition, Federal University of Bahia, Salvador, Brazil; e Department of Infectious Disease Epidemiology & International Health, London School of Hygiene & Tropical Medicine, London, United Kingdom

**Keywords:** Rapid weight gain, Body mass index, Growth trajectories, Birth weight

## Abstract

**Background:**

Obesity is considered a disease with negative health impacts at all life stages. Changes in growth patterns, such as postnatal rapid weight gain (RWG), can be important predictors of growth trajectories in children. We investigated the association between RWG during the first two years of life and subsequent BMI trajectories from the age 3–to 9 years, and whether the association differed by birth weight group.

**Methods:**

We used the data of a population-based cohort from the Cadastro Único (CadÚnico) of the Federal Government, the linkage of the National Live Births System (SINASC) and the National Food and Nutritional Surveillance System (SISVAN). The sample comprised 1.7 million Brazilian children aged from zero to nine years from 2008 to 2017. Mixed-effects models were used to estimate mean age-trajectories for BMI by RWG group.

**Findings:**

Children who experienced RWG during the first two years of life had higher mean BMI trajectories from 3 to 9 years, compared to those who did not. The difference was seen across all birth weight groups, and was more evident for the children with high birth weight. At age 9, the BMI difference between RWG and non-RWG children was 1.31 kg/m^2^ (boys) and 1.43 kg/m^2^ (girls) for children with adequate birth weight, 1.27 kg/m^2^ (boys) and 1.35 kg/m^2^ (girls) for low birth weight, and 2.25 kg/m^2^ (boys) and 2.86 kg/m^2^ (girls) for macrosomia.

**Interpretation:**

Children who experienced RWG during the first two years of life had higher BMI trajectories than children who did not. The finding highlighted the importance of monitoring child growth, which allows the early identification of potential growth deviations and the implementation of necessary interventions to ensure that children grow healthy and reach their full developmental potential.

**Funding:**

Coordenação de Aperfeiçoamento de Pessoal de Nível Superior—CAPES, CAPES/Print/UFBA; 10.13039/501100000765University College London (UCL); 10.13039/501100000272National Institute for Health Research (NIHR) Great Ormond Street Hospital Biomedical Research Centre; Fundação de Amparo à Pesquisa do Estado de Minas Gerais—FAPEMIG; 10.13039/501100003593National Council for Scientific and Technological Development—CNPq; CNPq/CGFP/DECIT/SECTICS; Departamento de Ciência e Tecnologia da Secretaria de Ciência, Tecnologia, Inovação e Complexo da Saúde do Ministério da Saúde; 10.13039/100010269Wellcome Trust.

**Research in context**

**Evidence before this study**

We searched PubMed and Web of Science for studies published from database inception to November 2024. We used a combination of Medical Subject Headings (MeSH) and free-text terms with the “AND” to combine the concepts of “birth weight”, “rapid weight gain”, and “BMI trajectories”. Rapid weight gain (RWG) in infancy has been consistently associated with higher body mass index (BMI) and increased risk of overweight and obesity in later childhood and adolescence. However, most studies have focused on single time-point outcomes, such as BMI or overweight status at preschool or school age. Studies that have evaluated how RWG influences longitudinal BMI trajectories are largely from high-income countries, with limited evidence from low- and middle-income settings, including Brazil. Additionally, few studies have explored this association stratified by birth weight groups, which may capture important differences in growth dynamics and long-term risk.

**Added value of this study**

This study adds new information about the association between rapid weight gain in the first two years of life and body mass index (BMI) trajectories during childhood, stratified by birth weight groups, in a large Brazilian cohort. Our analysis shows that children who experienced RWG in the first two years of life were more likely to follow higher-risk trajectories, including early-onset and persistently high BMI. We also demonstrate that the association between RWG and BMI trajectories from 3 to 9 years of age varies by birth weight category, suggesting that early growth patterns interact with perinatal factors to influence long-term growth outcomes.

**Implications of all the available evidence**

These findings reinforce the importance of monitoring growth throughout the first 1000 days of life as a critical window for identifying early signs of excessive weight gain and preventing unhealthy BMI trajectories. Birth weight and postnatal growth patterns play a central role in shaping long-term health. Strengthening growth surveillance during this period can be a key strategy to prevent both short- and long-term consequences of early rapid weight gain.

## Introduction

Child growth is a key indicator of children's health and development and reflects not only biological processes but also the social, nutritional and environmental conditions to which they are exposed to from the beginning of life.^1^^,^^2^ Among the factors that directly influence child growth, weight gain in early life has received increasing attention in the scientific literature. Rapid weight gain (RWG), particularly during the first two years of life, has been consistently associated with increased risk of overweight and obesity throughout childhood and the association persists to adulthood.^3^, ^4^, ^5^, ^6^ Moreover, RWG has been linked to adverse metabolic outcomes^7^ such as insulin resistance, hypertension and early cardiovascular disease.^8^ These findings highlight the “first thousand days” as a critical window for the prevention of chronic diseases throughout the life course.^2^

Despite the consensus on the risks associated with RWG, most studies of subsequent obesity have focused on BMI measurements at specific ages in childhood.^9^, ^10^, ^11^ This limits understanding of how growth trajectories evolve over time. In addition, most evidence originates from high-income countries,^12^ reducing its applicability to more diverse and unequal contexts, such as those in middle-income countries where multiple forms of nutritional insecurity coexist.^2^^,^^13^ Another important gap is that few studies have considered birth weight in the relationship between RWG and future growth, even though it is well established that children born with low birth weight tend to follow distinct postnatal growth patterns, including greater propensity for accelerated catch-up growth, which may have long-term implications for body composition and metabolic health.^3^^,^^14^^,^^15^

In light of these gaps, this study aimed to examine (1) the association between weight gain in the first two years of life and BMI trajectories from the age 3 to 9 years and (2) whether the association differed by birth weight group. Understanding how early weight gain influences BMI trajectories may contribute to identify critical periods for intervention and support efforts to prevent unhealthy growth patters during childhood.

## Methods

The population-based cohort used data extracted from the 100 million Brazilians Cohort.^16^ The baseline of this cohort was created using the Single Registry for Social Programs of the Federal Government (CadUnico). The CadUnico identifies and characterizes Brazilian families in situations of poverty and extreme poverty, defined as those with a monthly income of up to half a minimum wage per person or a total monthly income of up to three minimum wages.^17^ The database contains socioeconomic and demographic data of families and individuals.

To carry out this study, the baseline cohort was linked to the Live Birth Information System (SINASC) and the Food and Nutrition Surveillance System (SISVAN). The SINASC, implemented in 1990, now covers 97% of births nationwide.^18^ SINASC aims to collect comprehensive data on maternal characteristics and pregnancy, childbirth, and newborn conditions. This information was gathered through the Declaration of Live Birth, a mandatory national registration instrument feeding into the system. Additionally, the Food and Nutritional Surveillance System (SISVAN) is a system for monitoring the population's nutritional and dietary status, with a structural link to primary care across all stages of the life course in Brazil.^19^

### Linkage process

The linkage of three data sources was carried out as follows: initially, CadUnico (cohort baseline) was linked to SINASC using a non-deterministic method based on key attributes such as name, sex, children's date of birth, mother's name, and municipality of residence.^20^^,^^21^ Subsequently, the cohort baseline was linked to SISVAN through deterministic linkage via the Number Identification Social (NIS). For those without a NIS, a non-deterministic approach was employed using shared attributes like name, mother's name, date of birth, and sex. This process resulted in the formation of a cohort by merging three databases. CIDACS-RL tool, with an accuracy rate of 96%, was used for data linkage.^22^

The administrative database made available for is analysis was used following the regulations of the Brazilian National Research Ethics Commission. The present study is part of a larger study approved by the Research Ethics Committee of the Federal University of Minas Gerais under n° CAAE 37534620.3.0000.5149.

### Study population

**Fig. 1:**
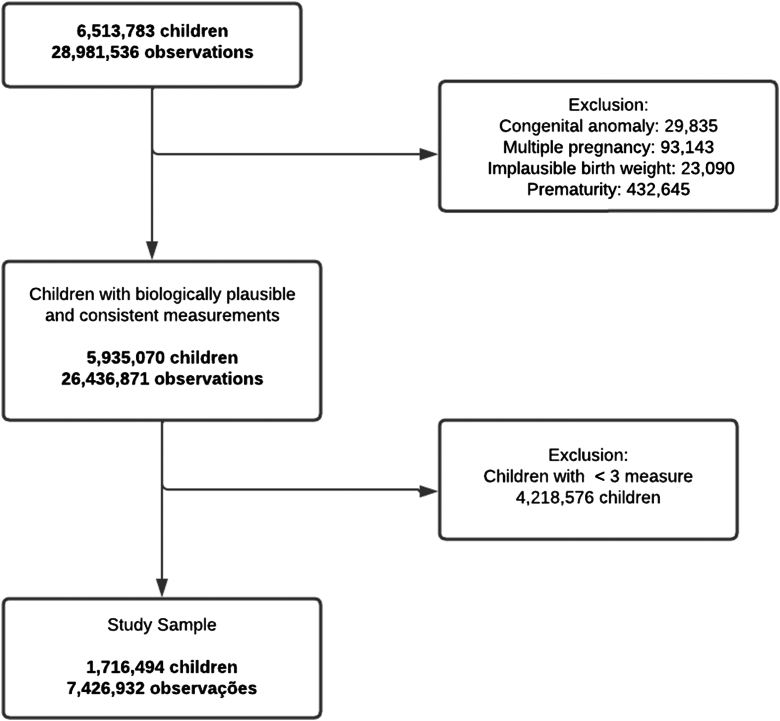
Study flowchart.

The study population included 1,716,494 children aged from 0 to 9 years with repeated weight measurements between 2008 and 2017 (total 7,426,932 measurements). Children were excluded if they had missing data on age, sex, weight, or height measurements. We also excluded those with congenital anomalies, implausible birth weights (<500 g or >6500 g), or implausible values based on WHO cutoffs for age- and sex-standardized z-scores for BMI (<−5 or >+5), height (<−6 or >+6), and weight (<−6 or >+5).^23^ Children with fewer than three repeated anthropometric measurements (weight or height) were also excluded. Children born preterm (<37 weeks) and from multiple pregnancies were also excluded from the analysis. Preterm children tend to exhibit postnatal growth patterns that differ from those of full-term children.^24^ Similarly, children from multiple pregnancies have distinct intrauterine growth environments and different postnatal growth trajectories.^25^ A detailed flowchart about the study population selection is provided in Fig. 1.

### Measures

We derived age- and sex-standardized z-scores for weight using WHO growth reference for under 5 years.^23^^,^^26^ We derived the difference between the last weight-for-age (WAZ) z-scores measurement before the age of 2 and the WAZ at birth. Rapid weight gain (RWG) was defined as the difference being greater than +0.67 standard deviations (SD).^6^^,^^11^ Weight (0.1 kg) was measured at each follow-up in the SISVAN^27^ and BMI (kg/m^2^) was calculated to construct mean BMI trajectories from 3 to 9 years of age.

The main characteristics considered for sample description were: sex, birth weight, residence area (urban; rural), marital status, maternal education, maternal race, maternal age (10–19 y; 20–34 y; 35–49 y), type of delivery (vaginal or cesarean). Birth weight was classified as low birth weight (<2500 g), adequate birth weight (≥2500 g and ≤4000 g) and macrosomia (>4000 g). Marital status was categorized as single, widow or divorced, married or in a stable union. Maternal education was categorized as none, 1–3, 4–7, 8 years or more. Self-declared maternal race was classified as white, black, Asian descent, pardo, indigenous. Residence area and race were obtained from CadUnico, while the remaining information was sourced from SINASC.

### Statistical analysis

We first applied mixed effects models to estimate the mean age-trajectories for BMI with 95% confidence intervals (CIs), from three to nine years for boys and girls separately. Random effect was used for individual specific intercepts. We explored fractional polynomials of age to capture the nonlinear curves for BMI trajectories. Based on the Akaike information criterion (AIC), Bayesian information criterion (BIC) and likelihood ratio test, the best fitting two-degree fractional polynomials included *age* and *age*^*2*^ (a quadratic function) for boys and girls. To examine the association between weight gain in the first 2 years and BMI trajectories across age, models were fitted by RWG status and included the interaction between RWG and age (as a fixed effect).

To examine whether the associations between RWG before age two and subsequent BMI trajectories differed by birth weight, models were also fitted for three birth weight groups: low birth weight (n = 63,589), adequate birth weight (n = 1,550,203) and macrosomia (n = 102,702) (Supplementary Table). To illustrate, we plotted mean BMI curves for children with and without RWG separately by birth weight groups. All analyzes were performed in the open access software R (for server version 4.1).

### Role of the funding source

The funder of the study had no role in the study design, data collection, data analysis, data interpretation, writing of the report or decision to submit.

## Results

Of the 1,716,494 children included in the study (52% girls [892,647] and 48% boys [823,775]), 33.1% [568,497] experienced RWG during the first two years of life. Table 1 presents maternal and child characteristics according to RWG status. The prevalence of low birth weight was much higher in the RWG (9.2% [52,499]) than non-RWG group (1.0% [11,090]). Compared to the non-RWG group, mothers in the RWG group were more likely to be from urban areas (70.5% vs 67% [400,484 vs 769,625]), had at least eight years of formal education (49.1% vs 43.8% [279,261 vs 502,915]), were also more likely to be White (32.7% vs 27.5 [185,707 vs 315,294]), were younger (<20 years: 19.9% vs 16.9% [112,908 vs 193,825]), and had caesarean delivery (40.8% vs 37.3% [231,894 vs 428,443]). For marriage status, there was little difference between RWG and non-RWG groups.

**Table 1:** Maternal and child characteristics by rapid weight gain (RWG) group, 2008–2017 (n = 1,716,494).

Variables	Total	non-RWG (%)	RWG (%)
	1,716,494 (100%)	1,147,997 (66.9)	568,497 (33.1)
**Child's sex**			
Boys	823,775 (48.0)	556,186 (48.5)	267,622 (47.1)
Girls	892,647 (52.0)	591,811 (51.5)	300,875 (52.9)
Missing	–	–	–
**Birth weight (g)**			
<2500 g	63,583 (3.7)	11,090 (1.0)	52,499 (9.2)
≥2500 g and ≤4000 g	1,550,142 (90.3)	1,041,132 (90.7)	509,071 (89.5)
>4000 g	102,697 (6.0)	95,775 (8.3)	6927 (1.2)
Missing	–	–	–
**Residence area**			
Urban	1,170,070 (68.2)	769,625 (67.0)	400,484 (70.5)
Rural	546,326 (31.8)	378,355 (33.0)	168,004 (29.5)
Missing	26 (0.0)	17 (0.0)	9 (0.0)
**Maternal marital status**			
Single/Widow/divorced	969,085 (56.5)	644,741 (56.2)	324,371 (57.1)
Married/stable union	716,803 (41.8)	482,691 (42.0)	234,150 (41.2)
Missing	30,541 (1.8)	20,565 (1.8)	9976 (1.7)
**Maternal education (y)**			
None	36,536 (2.1)	27,229 (2.4)	9307 (1.6)
1–3	195,612 (11.4)	139,745 (12.2)	55,867 (9.8)
4–7 years	657,112 (38.3)	448,069 (39.0)	209,043 (36.8)
8 years or more	782,176 (45.6)	502,915 (43.8)	279,261 (49.1)
Missing	45,058 (2.6)	30,039 (2.6)	15,019 (2.6)
**Maternal race**			
White	501,001 (29.2)	315,294 (27.5)	185,707 (32.7)
Black	61,374 (3.6)	39,692 (3.5)	21,282 (3.8)
Asian descent	6570 (0.4)	4427 (0.4)	2143 (0.4)
Pardo	1,131,614 (65.9)	776,202 (67.6)	335,413 (62.5)
Indigenous	15,921 (0.9)	12,372 (1.1)	3549 (0.6)
Missing	13 (0.0)	10 (0.0)	3 (0.0)
**Maternal age (y)**			
<20	306,733 (17.9)	193,825 (16.9)	112,908 (19.9)
20–34	1,246,436 (72.6)	844,508 (73.6)	401,928 (70.7)
≥35	163,217 (9.5)	109,588 (9.5)	53,629 (9.4)
Missing	108 (0.0)	76 (0.0)	32 (0.0)
**Type of delivery**			
Vaginal	1,052,464 (61.3)	717,105 (62.5)	335,359 (59.0)
Cesarean	660,337 (38.5)	428,443 (37.3)	231,894 (40.8)
Missing	3693 (0.22)	2449 (0.2)	1244 (0.2)

Note: All RWG vs non-RWG comparisons: Pearson's chi-square test, p < 0.001.

**Fig. 2:**
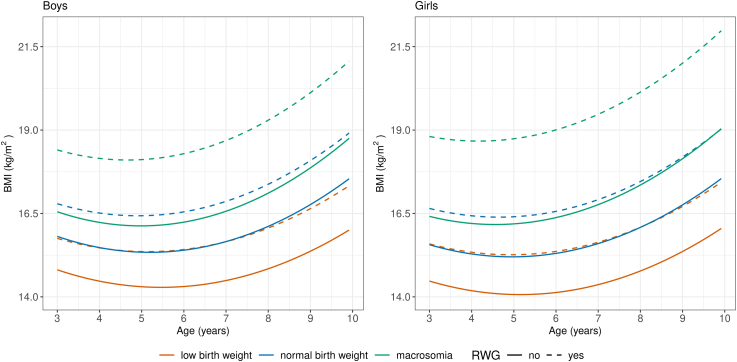
Trajectories of body mass index (BMI, kg/m^2^) from ages 3 to 9 years in children who did or did not rapid weight gain during the first two years of life, stratified by birthweight groups.

Fig. 2 shows that in general, the mean BMI trajectory decreased after age 3, reaching an adiposity rebound (around age 5), and subsequently increased until age 9. Children who experienced rapid weight gain during the first two years of life exhibited higher mean BMI trajectories from 3 to 9 years compared to those who did not. This pattern was observed across all birth weight groups.

The difference in mean BMI tended to increase with age for all birth weight groups. For example, for the adequate birth weight group, the difference was 0.97 kg/m^2^ (boys) and 1.09 kg/m^2^ (girls) at 3 years, increased to 1.31 kg/m^2^ and 1.43 kg/m^2^ respectively at 9 years. For the low birth weight group, the respective difference was 0.94 kg/m^2^ (boys) and 1.11 kg/m^2^ (girls) at 3 years, 1.27 kg/m^2^ and 1.35 kg/m^2^ at 9 years, respectively. However, the respective difference was greatest for the macrosomia group, 1.85 kg/m^2^ (boys) and 2.39 kg/m^2^ (girls) at 3 years, increased to 2.25 kg/m^2^ and 2.86 kg/m^2^ respectively at 9 years (Table 2).

**Table 2:** Mean BMI (kg/m^2^) and 95% CIs and difference (d) by age for boys and girls who did and did not experience rapid weight gain (RWG) in the first 2 years of life by birth weight group (n = 1,716,494).

Age (y)	Low birth weight					Adequate birth weight					Macrosomia				
	non- RWG		RWG		d^a^	non- RWG		RWG		d^a^	non- RWG		RWG		d^a^
	Mean	(95% CIs)	Mean	(95% CIs)		Mean	(95% CIs)	Mean	(95% CIs)		Mean	(95% CIs)	Mean	(95% CIs)	
Boys															
3	14.81	(14.63–14.99)	15.75	(15.57–15.94)	0.94	15.81	(15.78–15.84)	16.79	(16.76–16.81)	0.97	16.55	(16.42–16.68)	18.40	(18.27–18.53)	1.85
4	14.47	(14.29–14.65)	15.47	(15.28–15.65)	0.99	15.48	(15.45–15.51)	16.51	(16.48–16.54)	1.03	16.23	(16.10–16.36)	18.15	(18.02–18.28)	1.92
5	14.30	(14.12–14.49)	15.36	(15.17–15.54)	1.05	15.34	(15.31–15.37)	16.43	(16.40–16.46)	1.09	16.13	(16.00–16.26)	18.11	(17.98–18.24)	1.99
6	14.31	(14.13–14.49)	15.42	(15.24–15.60)	1.11	15.40	(15.37–15.43)	16.54	(16.51–16.57)	1.15	16.24	(16.11–16.37)	18.29	(18.16–18.42)	2.05
7	14.48	(14.30–14.67)	15.65	(15.47–15.83)	1.16	15.66	(15.63–15.69)	16.86	(16.83–16.89)	1.21	16.57	(16.44–16.70)	18.69	(18.56–18.82)	2.11
8	14.84	(14.66–15.03)	16.06	(15.88–16.25)	1.22	16.11	(16.08–16.14)	17.37	(17.34–17.40)	1.26	17.11	(16.98–17.24)	19.29	(19.16–19.42)	2.19
9	15.36	(15.18–15.55)	16.65	(16.46–16.83)	1.27	16.77	(16.74–16.80)	18.09	(18.06–18.12)	1.31	17.87	(17.74–17.99)	20.12	(19.99–20.25)	2.25
Girls															
3	14.47	(14.33–14.61)	15.59	(15.45–15.73)	1.11	15.56	(15.53–15.58)	16.65	(16.62–16.67)	1.09	16.41	(16.22–16.60)	18.80	(18.60–18.99)	2.39
4	14.18	(14.04–14.32)	15.34	(15.20–15.48)	1.15	15.29	(15.26–15.31)	16.43	(16.40–16.45)	1.14	16.20	(16.00–16.39)	18.67	(18.48–18.86)	2.47
5	14.07	(13.93–14.21)	15.26	(15.12–15.40)	1.19	15.20	(15.17–15.22)	16.40	(16.37–16.42)	1.20	16.19	(15.99–16.38)	18.73	(18.54–18.92)	2.55
6	14.13	(13.99–14.27)	15.36	(15.22–15.50)	1.23	15.30	(15.27–15.32)	16.56	(16.53–16.58)	1.26	16.37	(16.18–16.56)	19.00	(18.81–19.19)	2.63
7	14.37	(14.23–14.51)	15.64	(15.50–15.78)	1.27	15.60	(15.57–15.62)	16.91	(16.88–16.93)	1.32	16.76	(16.57–16.95)	19.47	(19.28–19.66)	2.71
8	14.78	(14.63–14.92)	16.09	(15.95–16.23)	1.31	16.08	(16.05–16.10)	17.46	(17.43–17.48)	1.37	17.35	(17.16–17.54)	20.14	(19.95–20.33)	2.78
9	15.36	(15.22–15.50)	16.71	(16.57–16.85)	1.35	16.76	(16.73–16.78)	18.19	(18.16–18.21)	1.43	18.14	(17.95–18.33)	21.01	(20.82–21.20)	2.86

a Difference in mean BMI (kg/m^2^) between children did and did not experience RWG.

## Discussion

In this large population cohort of children under 10 years old, we found that rapid weight gain (RWG) in the first 2 years of life was associated with higher BMI trajectories of BMI from 3 to 9 years. The difference in mean BMI widened with age in all birth weight groups, indicating that the association between RWG persistened with increasing throughout childhood. The pattern was particularly evident for children with macrosomia.

The association between RWG and obesity is well-documented in many populations.^5^^,^^9^^,^^10^^,^^28^^,^^29^ Various previous studies have shown that RWG is associated with overweight and obesity in both the short and long term.^28^^,^^29^ A study conducted in four Latin American countries showed that the risk of obesity for children born with normal weight who experienced RWG, compared to those without RWG, was 6.1 times greater in Brazil, 4.4 times greater in Bolivia, 6.7 times greater in Colombia, and 12.2 times greater in Peru.^10^ Another study conducted with low-income families found that children who experienced RWG during the first year of life had almost ten times the chance of becoming obese by the age of three.^30^ Research has also shown correlations between RWG and other adiposity indices, such as a higher percentage of body fat, higher waist-to-height ratio,^31^ greater waist circumference,^28^ and increased abdominal and visceral fat.^5^ RWG has also been associated with a higher cardiometabolic risk.^32^ In our study, among children who exhibited rapid weight gain (RWG) during the first two years of life, 18.62% were classified as overweight and 6.77% as obese. In contrast, among those who did not present RWG, 7.8% were classified as overweight and 2.12% as obese (data not show).

The evidence already produced focuses on obesity at specific ages, therefore it cannot capture the differences that occur in growth over time. BMI growth changes with age.^33^ BMI increases from birth until it reaches a peak at seven months of age; then decreases until reaching the lowest BMI point between five and seven years (i.e. adiposity rebound), and progressively increases until adolescence.^33^^,^^34^ These growth patterns characterize a normal BMI evolution trajectory of children. Deviations from this trajectory may lead to obesity and excessive body fat accumulation.^35^ For example, an earlier age at adiposity rebound is associated with an increased risk of obesity.^34^ Thus relevant to devise prevention and intervention strategies specific to each age period.

Among the few existing studies that show an association between RWG and BMI trajectories, a notable study conducted with British children highlighted that RWG occurring in the first three years of life was associated with a higher average BMI trajectory from ages 5 to 14. In the British cohort, the magnitude of the average BMI differences were also found to increase with age, from 0.76 kg/m^2^ in boys and 0.87 kg/m^2^ in girls at 5 years, to 1.37 kg/m^2^ and 1.75 kg/m^2^ at 14 years respectively. The differences remained even after adjustment for important potential confounders.^12^ Studies using standardized BMI indices have shown that children with RWG consistently had higher standardized weight scores than children without RWG. A study of Spanish children found that those had RWG during the first six months of life had higher BMI z-score trajectories from one to six years of age.^36^ In Germany, term-born children with adequate birth weight who experienced rapid weight gain (RWG) exhibited higher average BMI z-score trajectories from two to seven years of age.^37^

Additionally, our study used a large population sample, which made it possible to evaluate BMI trajectories for different groups at birth. Children born with low birth weight are more likely to experience rapid weight gain in early life as a compensatory growth response.^5^ Scientific evidence highlights that, in the short term, this growth may be beneficial.^14^^,^^38^ However, in the long term, it may lead to deleterious health effects, such as higher body mass index (BMI) and elevated cholesterol levels.^14^ The combination of high birth weight and rapid weight gain in the first years of life is strongly associated with an increased risk of later obesity.^39^ High birth weight partly reflects greater fetal exposure to nutrients, which may lead to the development of a metabolic environment that favors fat accumulation.^40^ Children with high birth weight may maintain a pattern of rapid growth in the early years of life, which may increase the risk of childhood obesity and later metabolic disturbances, such as diabetes and dyslipidemia.^41^^,^^42^

Therefore, monitoring infant growth is essential to identify patterns of rapid weight gain and guide early interventions aimed at promoting healthy growth and potentially reducing the risk of obesity. The first thousand days of a child's life, during which RWG is most frequently observed, represent a crucial window for monitoring growth and supporting interventions to help children achieve their growth potential.

### Strengths and limitations

An important strength of this study is the longitudinal, population-based design, with a sufficiently large sample size that provides the power to analyze growth patterns over time stratified by birth weight, rather than relying on cross-sectional analysis or a longitudinal data with limited sample size. Another potential strength was the use of the mixed-effects model, which can accommodate complex data structures and analyze irregular data, providing robust estimates. However, limitations exist. First, due to the irregular nature of the data, children's weight measurements were not collected at the same times. We used the last available anthropometric measurement before 24 months of age as a proxy for the 2-year measurement. Although this may introduce some measurement variability due to differences in the exact age at assessment, it allowed for the inclusion of more participants and better captured the cumulative effect of early weight gain. Second, as this is an observational study, we cannot exclude the possibility of residual confounding, and no formal causal inference was attempted. Future studies using more with adjustment strategies or fully adjusted are warranted to further clarify these associations. Finally, the birth cohort consists of children from families living in poverty and extreme poverty in Brazil. Therefore, the results should be interpreted cautiously considering these points.

Research on weight trajectories in children highlights critical periods of early years that may influence the development of excess adiposity across the life course. BMI typically increases from birth, peaking between six and twelve months of age. It then declines, reaching its lowest point between five and six years, before progressively rising again through adolescence.^33^^,^^34^ The BMI trajectory observed in this study aligns closely with patterns described in the literature, which reinforces the representativeness, reliability, and external validity of our findings—an anticipated outcome given the large size of the studied population. Moreover, this alignment underscores the robustness of the cohort composition methods, achieved through linkage across three administrative databases. These growth patterns not only provide a benchmark for tracking normal weight trajectories but also emphasize the importance of identifying deviations, as these can signal risks for obesity and excess fat accumulation.^35^

### Conclusion

This study demonstrated that children who experienced rapid weight gain (RWG) in early life presented higher average BMI trajectories throughout childhood compared to those who did not experience RWG. This pattern presented across birth weight groups. These findings support that weight gain during the first two years of life is associated with trajectories until 9 years, and that higher weight gain in this period may be linked to increased risk of overweight and obesity in later childhood. Given the long-term impact of RWG on child growth, the results reinforce the importance of early identification and monitoring of weight trajectories during infancy and early childhood. From a public health perspective, these findings highlight the critical importance of strengthening existing strategies for early growth monitoring and timely intervention, particularly those aimed at preventing excessive weight gain during the first two years of life.

## Contributors

CSV and GVM designed the study. CSV wrote the first version. CSV and JFMS verified the data, had access to raw data and analyzed the data. LL, RCRS, ESP, MLB and GVM provided critical feedback regarding the analyses and revised the manuscript. All authors approved the final manuscript and accepted responsibility for the decision to submit for publication.

## Data sharing statement

All data supporting this study were obtained from the Center for Data and Knowledge Integration for Health (CIDACS). These were licensed for exclusive use in the present study and, due to the privacy rules of the Brazilian Ethics Committee, are not openly available. Upon request with adequate justification and approval of an ethics committee, controlled access to data is considered and, if possible, allowed access.

## Declaration of interests

We declare no competing interests.
