# Supplementary material for: Rapid weight gain in first 2 years of life and BMI trajectories from 3 to <10 years: a population-based longitudinal study of 1.7 million Brazilian children
Source: Lancet Reg Health Am. 2025 Dec 3;54:101326. doi: 10.1016/j.lana.2025.101326 (PMC12719978; doi:10.1016/j.lana.2025.101326)
Supplement: Supplementary Table [file mmc1.pdf]

Supplementary Table

Table S1 - Estimated Coefficients for BMI (kg/m²) in a group of low birthweight, by sex*

	Random effects	*β*	EP	p value
**Boys**				
Intercept	-	16.870471	0.07547803	<0.001
Age (months)	-	-0.078855	0.00204144	<0.001
Age^2 (months)	-	0.000601	0.00001475	<0.001
RWG (yes)	-	0.776201	0.05554493	<0.001
RWG*age		0.004631	0.00085637	<0.001
σ intercept	1.455492	-	1.611944	-
σ residual	1.312614	-	1.368846	-
AIC	399358.4	-	-	-
BIC	399425.4	-	-	-
**Girls**				
Intercept	-	16.389825	0.05734632	<0.001
Age (months)	-	-0.075059	0.00144059	<0.001
Age^2 (months)	-	0.000607	0.00000954	<0.001
RWG (yes)	-	0.994796	0.04275605	<0.001
RWG*age		0.003290	0.00059519	<0.001
σ intercept		-	1.846964	-
σ residual		-	1.554677	-
AIC	693501.6	-	-	-
BIC	693572.1	-	-	-

*Using Mixed Effects Models

Table S2 - Estimated Coefficients for BMI (kg/m²) in adequate birthweight child, by sex.*

	Random effects	*β*	EP	p value
**Boys**				
Intercept	-	18.005458	0.011343136	<0.001
Age (months)	-	-0.085647	0.000356765	<0.001
Age^2 (months)	-	0.000687	0.000002741	<0.001
RWG (yes)	-	0.809115	0.008202409	<0.001
RWG*age	-	0.004725	0.000127006	<0.001
σ intercept		-	1.611944	-
σ residual		-	1.368846	-
AIC	11742167	-	-	-
BIC	11742257	-	-	-
**Girls**				
Intercept	-	17.539468	0.010326688	<0.001
Age (months)	-	-0.078692	0.000301404	<0.001
Age^2 (months)	-	0.000662	0.000002114	<0.001
RWG (sim)	-	0.912614	0.007864647	<0.001
RWG*age	-	0.004817	0.000110263	<0.001
σ intercept		-	1.846964	-
σ residual		-	1.554677	-
AIC	14598690	-	-	-
BIC	14598782	-	-	-

*Using Mixed Effects Models

Table S3 - Estimated Coefficients for BMI (kg/m²) in macrosomia child, by sex.*

	Random effects	*β*	EP	p value
**Boys**				
Intercept	-	18.800673	0.03934180	<0.001
Age (months)	-	-0.089418	0.00126058	<0.001
Age^2 (months)	-	0.000748	0.00000974	<0.001
RWG (yes)	-	1.652140	0.05274959	<0.001
RWG*age	-	0.005568	0.00082409	<0.001
σ intercept		-	1.611944	-
σ residual		-	1.368846	-
AIC	1013117	-	-	-
BIC	1013190	-	-	-
**Girls**				
Intercept	-	18.250358	0.04780737	<0.001
Age (months)	-	-0.076070	0.00142133	<0.001
Age^2 (months)	-	0.000695	0.00001003	<0.001
RWG (sim)	-	2.158608	0.08506213	<0.001
RWG*age	-	0.006535	0.00119008	<0.001
σ intercept		-	1.846964	-
σ residual		-	1.554677	-
AIC	731742	-	-	-
BIC	731812.6	-	-	-

*Using Mixed Effects Models
